# *S*-2-Amino-5-(dimethyl­ammonio)phenyl sulfothio­ate

**DOI:** 10.1107/S1600536809016158

**Published:** 2009-05-07

**Authors:** Gordana Pavlović, Livio Racané, Vesna Tralić-Kulenović

**Affiliations:** a Faculty of Textile Technology, Laboratory of Applied Chemistry, University of Zagreb, Prilaz baruna Filipovića 28a, HR-10000 Zagreb, Croatia

## Abstract

The title compound, C_8_H_12_N_2_O_3_S_2_, has been isolated as an inter­mediate in the synthesis of methyl­ene blue dye, the best known phenothia­zine dye, and structurally characterized as a zwitterion. The crystal structure is dominated by inter­molecular N—H⋯O hydrogen bonds between the amine and sulfothio­ate groups, with graph-set motif *C*(9)*R*_2_^2^(8), involving anti­parallel chains and a centrosymmetric eight-membered ring. A hydrogen bond with graph-set motif *R*_2_^2^(14) between the ammonium and sulfothio­ate groups completes the two-dimensional network in the *ab* plane. Inter­molecular C—H⋯O hydrogen bonds are also present in the crystal.

## Related literature

For methyl­ene blue dye, see: Bernthasen (1889 ▶); Zollinger (1991 ▶); Hunger (2003 ▶). For its preparation, see: Leventis *et al.* (1997 ▶). For the synthesis of the title compound, see: Bogert & Updike (1927 ▶); Bennett & Bell (1943 ▶). For bond-length data, see: Trinajstić (1968 ▶); Allen *et al.* (1987 ▶).
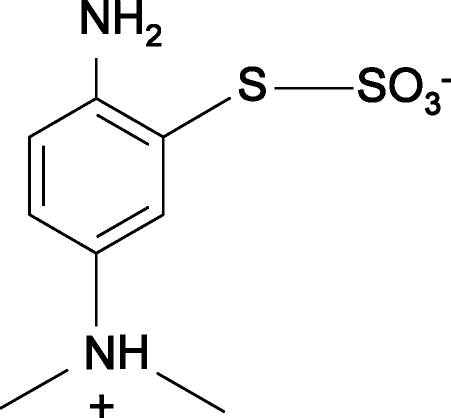


## Experimental

### 

#### Crystal data

C_8_H_12_N_2_O_3_S_2_
*M* *_r_* = 248.32
Monoclinic, 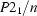

*a* = 12.0593 (1) Å
*b* = 7.3651 (1) Å
*c* = 12.2312 (1) Å
β = 95.0766 (8)°
*V* = 1082.09 (2) Å^3^
*Z* = 4
Cu *K*α radiation
μ = 4.41 mm^−1^
*T* = 296 K
0.48 × 0.37 × 0.29 mm

#### Data collection

Xcalibur Nova diffractometer with enhance (Cu) X-ray source and Onyx CCD
Absorption correction: multi-scan (*CrysAlis RED*; Oxford Diffraction, 2008 ▶) *T*_min_ = 0.628, *T*_max_ = 1.000 (expected range = 0.175–0.279)
5132 measured reflections
2153 independent reflections
2006 reflections with *I* > 2σ(*I*)
*R*_int_ = 0.015

#### Refinement

*R*[*F* ^2^ > 2σ(*F* ^2^)] = 0.034
*wR*(*F* ^2^) = 0.092
*S* = 1.07
2153 reflections
150 parameters
H atoms treated by a mixture of independent and constrained refinement
Δρ_max_ = 0.33 e Å^−3^
Δρ_min_ = −0.19 e Å^−3^

### 

Data collection: *CrysAlis CCD* (Oxford Diffraction, 2008 ▶); cell refinement: *CrysAlis RED* (Oxford Diffraction, 2008 ▶); data reduction: *CrysAlis RED*; program(s) used to solve structure: *SHELXL97* (Sheldrick, 2008 ▶); program(s) used to refine structure: *SHELXL97* (Sheldrick, 2008 ▶); molecular graphics: *ORTEP-3 for Windows* (Farrugia, 1997 ▶) and *Mercury* (Macrae *et al.*, 2008 ▶); software used to prepare material for publication: *SHELXL97*.

## Supplementary Material

Crystal structure: contains datablocks I, global. DOI: 10.1107/S1600536809016158/kp2216sup1.cif

Structure factors: contains datablocks I. DOI: 10.1107/S1600536809016158/kp2216Isup2.hkl

Additional supplementary materials:  crystallographic information; 3D view; checkCIF report

## Figures and Tables

**Table 1:** Hydrogen-bond geometry (Å, °)

*D*—H⋯*A*	*D*—H	H⋯*A*	*D*⋯*A*	*D*—H⋯*A*
N1—H11*N*⋯O1^i^	0.87 (3)	2.36 (3)	3.136 (3)	148 (2)
N1—H21*N*⋯O1^ii^	0.82 (2)	2.28 (2)	3.010 (2)	148 (2)
N2—H12*N*⋯O3^iii^	0.88 (2)	1.89 (2)	2.769 (2)	175 (2)
C5—H5⋯O3^i^	0.93	2.55	3.376 (2)	148
C8—H8*A*⋯O2^iv^	0.96	2.41	3.209 (3)	141

Symmetry codes: (i) 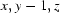; (ii) 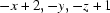; (iii) 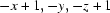; (iv) 
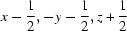
.

**Fig. 1.**
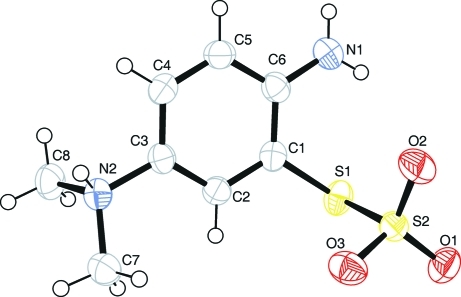
The molecular structure of (I) with the atom numbering scheme. Displacement ellipsoids are drawn at the 50% probability level.

**Fig. 2.**
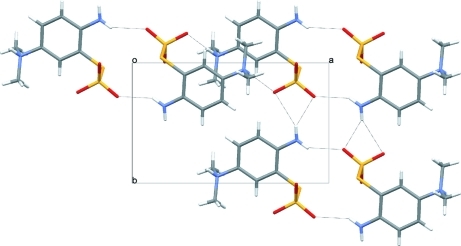
Crystal structure of (I) viewed down the c axis. Hydrogen bonds are drawn by dashed lines.

**Fig. 3.**
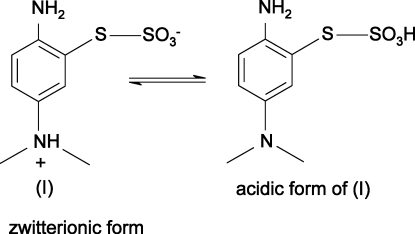
The zwitterionic and acid forms of the title compound.

## supplementary crystallographic information

### Comment

Phenothiazine dyes are class of colorants with application in various fields of which the methylene blue is the most well known (Zollinger, 1991; Hunger, 2003). Commercially, methylene blue is produced by oxidation of 4-*N*,*N*-dimethylaminoaniline with Na_2_Cr_2_O_7_ in the presence of Na_2_S_2_O_3_, followed by the further oxidation in the presence of *N*,*N*-dimethylaniline, usually without isolation of intermediate 4-*N*,*N*-dimethylaminoaniline-2-tiosulfuric acid (Leventis *et al.*, 1997). Namely, this compound was first described in 1889 (Bernthasen, 1889) and in the last hundred years reported by several authors. Moreover, in the literature the compound is described as phenyl *O*-hydrogen sulfothioate acid but the possibility of zwitterionic form (I) (Scheme 1) was not reported. Following one of the known method for preparation of 4-*N*,*N*-dimethylaminoaniline-2-tiosulfuric acid (Bogert & Updike, 1927), we isolated *S*-2-amino-5-(dimethylammonio)phenyl sulfothioate (I) determined by single-crystal structure analysis (Scheme, Fig. 1).

The single S—S bond distance value of sulfothioate group is 2.0985 (5) Å. The S—C bond in (I) is 1.768 (2) Å, reflecting aprox. 20% of π bond character according to N. Trinajstić (Trinajstić, 1968). The C_ar_—N bond formed by amine group has significantπ character (1.360 (3) Å). On the contrary, C—N bonds of the *N*,*N*-dimethylammonio groups are essentially single bonds (N2—C7 1.491 (2) Å and N2—C8 1.501 (2) Å). The values observed are in accordance with the literature data (Allen *et al.*, 1987).

The relative orientation of the sulfothioate group to the phenyl ring is defined by the torsion angle S2—S1—C3—C4 (92.93 (13)°). The twist around Car-Nsp3 bond is described by C_ar_—C_ar_—Nsp^3^—Csp^3^ torsion angle of 73.97 (18)° (for the atom sequence C6—C1—N2—C8).

The rather complex hydrogen bond network in (I) (Table 1, Fig. 2) is characterized by the N—H···O and the C—H···O intermolecular hydrogen bonds. The atom N1 acts as double proton donor and the atoms O1 and O3 as double proton acceptors (Table 1). The C—H···O intermolecular hydrogen bonds are formed between Car-H groups along with the C8 atom of 5-*N*,*N*-dimethylammonio cation and O atoms of S—SO_3_^-^ fragment.

At the unitary level antiparallel infinite chains are formed by the N1—H11N···O1^i^ (i = *x*, -*y*, *z*) hydrogen bonds between amino and sulfothioate groups (Table 1, Fig. 2). The R22(8) rings are formed *via* N1—H11N···O1^i^ (i = *x*, -*y*, *z*) and N1—H21N···O1^ii^ (ii = 2 - *x*, -*y*, 1 - *z*) hydrogen bonds, thus N1 amino group participates in bifurcated hydrogen bond. The combination of these two primary motifs, chain and ring, generates a new 14-membered ring of the second level of graph-set notation: *N*2=R22(14) involving N+2-H···O3 hydrogen bond. Consequently, the crystal structure can be described as the two-dimensional-network in the (*ab*) plane.

### Experimental

*N*,*N*-dimethylaniline was dissolved in aqueous HCl and nitrosilated with NaNO_2_ (Bennett & Bell, 1943). The resulting crude 4-nitroso-*N*,*N*-dimethylaniline hydrochloride was isolated and dissolved in aqueous acetic acid. The cold water solution of Na_2_S_2_O_3_ was added and the reaction mixture was stirred at 273 - 278 K for several h (Bogert & Updike, 1927), and left for two days at room temperature. The crude product was filtered off, and crystallized from water. The obtained crystals of *S*-2-amino-5-(dimethylammonium)phenyl sulfothioate (I) were in the form of blue prisms. Spectroscopic analysis, IR (ATR, cm^-1^): 3451 (*m*), 3342 (*m*), 3034 (w), 2657 (*m*), 1616 (*s*), 1504 (*s*), 1458 (*m*), 1400 (*m*), 1319 (w), 1242 (*s*), 1161 (*s*), 1134 (*s*), 1003 (*s*), 906 (*m*), 880 (w), 822 (*m*), 675 (w), 622 (*s*), 544 (*m*). ^1^H NMR (300 MHz, DMSO-d_6_):δ 8.99 (br s, 2H), 7.18 (s, 1H), 7.10–7.03 (m, 2H), 2.99 (s, 6H). Analysis, calculated for C_8_H_12_N_2_O_3_S_2_: C 38.69, H 4.87, N 11.28%; found: C 38.65, H 4.91, N 11.21%.

### Refinement

Hydrogen atoms bonded to the nitrogen atoms of amino and ammonio groups were found in the difference Fourier electron-density maps and refined freely. All hydrogen atoms attached to the carbon atoms were generated at calculated positions and refined by applying the riding model (*U*_iso_ (H) = 1.2 *U*_eq_ (C) and Csp2-H distance 0.93 Å; Csp3-H 0.96 Å and *U*_iso_ (H) = 1.5 *U*_eq_ (C).

### Crystal data

**Table:**

C_8_H_12_N_2_O_3_S_2_	*F*(000) = 520
*M**_r_* = 248.32	*D*_x_ = 1.524 Mg m^−^^3^
Monoclinic, *P*2_1_/*n*	Cu *K*α radiation, λ = 1.54184 Å
*a* = 12.0593 (1) Å	Cell parameters from 4267 reflections
*b* = 7.3651 (1) Å	θ = 3.6–76.1°
*c* = 12.2312 (1) Å	µ = 4.41 mm^−^^1^
β = 95.0766 (8)°	*T* = 296 K
*V* = 1082.09 (2) Å^3^	Prism, blue
*Z* = 4	0.48 × 0.37 × 0.29 mm

### Data collection

**Table:**

κ geometry Xcalibur Nova diffractometer with enhance (Cu) X-ray source and Onyx CCD	2153 independent reflections
Radiation source: fine-focus sealed tube	2006 reflections with *I* > 2σ(*I*)
graphite	*R*_int_ = 0.015
Detector resolution: 10.4323 pixels mm^-1^	θ_max_ = 75.0°, θ_min_ = 4.9°
Enhance (Cu) X–ray Source scans	*h* = −13→15
Absorption correction: multi-scan (*CrysAlis RED*; Oxford Diffraction, 2008)	*k* = −9→5
*T*_min_ = 0.628, *T*_max_ = 1.000	*l* = −15→14
5132 measured reflections	

### Refinement

**Table:**

Refinement on *F*^2^	Primary atom site location: structure-invariant direct methods
Least-squares matrix: full	Secondary atom site location: difference Fourier map
*R*[*F*^2^ > 2σ(*F*^2^)] = 0.034	Hydrogen site location: inferred from neighbouring sites
*wR*(*F*^2^) = 0.092	H atoms treated by a mixture of independent and constrained refinement
*S* = 1.07	*w* = 1/[σ^2^(*F*_o_^2^) + (0.0551*P*)^2^ + 0.3077*P*] where *P* = (*F*_o_^2^ + 2*F*_c_^2^)/3
2153 reflections	(Δ/σ)_max_ < 0.001
150 parameters	Δρ_max_ = 0.33 e Å^−^^3^
0 restraints	Δρ_min_ = −0.18 e Å^−^^3^

### Special details

**Table:**

Experimental. Empirical absorption correction using spherical harmonics, implemented in SCALE3 ABSPACK scaling algorithm.

### Fractional atomic coordinates and isotropic or equivalent isotropic displacement parameters (Å^2^)

**Table:**

	*x*	*y*	*z*	*U*_iso_*/*U*_eq_	
S1	0.85191 (3)	0.04337 (6)	0.62282 (3)	0.04476 (15)	
S2	0.83204 (3)	0.15312 (5)	0.46396 (3)	0.03773 (14)	
O1	0.92392 (11)	0.27914 (18)	0.46826 (12)	0.0521 (3)	
O2	0.83442 (12)	0.00876 (19)	0.38531 (12)	0.0556 (3)	
O3	0.72379 (11)	0.24407 (18)	0.45543 (11)	0.0506 (3)	
N1	0.83654 (15)	−0.3588 (3)	0.56480 (16)	0.0531 (4)	
H11N	0.832 (2)	−0.469 (4)	0.539 (2)	0.060 (7)*	
H21N	0.891 (2)	−0.295 (3)	0.556 (2)	0.057 (7)*	
N2	0.43258 (11)	−0.05829 (19)	0.68128 (11)	0.0369 (3)	
H12N	0.3836 (17)	−0.113 (3)	0.6345 (17)	0.042 (5)*	
C1	0.53946 (13)	−0.1301 (2)	0.64861 (12)	0.0354 (3)	
C2	0.63340 (13)	−0.0239 (2)	0.65023 (13)	0.0371 (3)	
H2	0.6306	0.0971	0.6715	0.045*	
C3	0.73290 (13)	−0.0974 (2)	0.62006 (13)	0.0375 (3)	
C4	0.73946 (14)	−0.2821 (2)	0.59014 (13)	0.0391 (3)	
C5	0.64152 (14)	−0.3866 (2)	0.59039 (14)	0.0413 (4)	
H5	0.6433	−0.5087	0.5712	0.050*	
C6	0.54366 (14)	−0.3119 (2)	0.61841 (13)	0.0388 (3)	
H6	0.4799	−0.3832	0.6172	0.047*	
C7	0.42090 (16)	0.1431 (2)	0.67521 (17)	0.0491 (4)	
H7A	0.4696	0.1979	0.7322	0.074*	
H7B	0.3453	0.1762	0.6845	0.074*	
H7C	0.4403	0.1848	0.6050	0.074*	
C8	0.40933 (16)	−0.1240 (3)	0.79311 (15)	0.0491 (4)	
H8A	0.4120	−0.2543	0.7948	0.074*	
H8B	0.3368	−0.0838	0.8091	0.074*	
H8C	0.4643	−0.0760	0.8470	0.074*	

### Atomic displacement parameters (Å^2^)

**Table:**

	*U*^11^	*U*^22^	*U*^33^	*U*^12^	*U*^13^	*U*^23^
S1	0.0369 (2)	0.0545 (3)	0.0423 (2)	−0.01012 (16)	0.00067 (16)	0.00048 (17)
S2	0.0361 (2)	0.0368 (2)	0.0409 (2)	−0.00114 (14)	0.00620 (15)	−0.00381 (14)
O1	0.0461 (7)	0.0505 (7)	0.0602 (8)	−0.0109 (6)	0.0076 (6)	0.0049 (6)
O2	0.0643 (8)	0.0537 (8)	0.0503 (7)	0.0016 (6)	0.0127 (6)	−0.0157 (6)
O3	0.0435 (7)	0.0476 (7)	0.0594 (8)	0.0071 (5)	−0.0029 (5)	−0.0075 (6)
N1	0.0463 (9)	0.0465 (9)	0.0690 (11)	0.0051 (7)	0.0192 (8)	−0.0029 (8)
N2	0.0344 (7)	0.0419 (7)	0.0347 (7)	−0.0012 (5)	0.0046 (5)	−0.0001 (5)
C1	0.0344 (7)	0.0402 (8)	0.0320 (7)	0.0011 (6)	0.0046 (6)	0.0004 (6)
C2	0.0394 (8)	0.0361 (8)	0.0363 (7)	−0.0021 (6)	0.0060 (6)	−0.0030 (6)
C3	0.0354 (7)	0.0425 (8)	0.0348 (7)	−0.0034 (6)	0.0047 (6)	−0.0003 (6)
C4	0.0415 (8)	0.0425 (9)	0.0336 (7)	0.0026 (7)	0.0060 (6)	0.0006 (6)
C5	0.0487 (9)	0.0349 (8)	0.0409 (8)	−0.0005 (7)	0.0067 (7)	−0.0016 (6)
C6	0.0394 (8)	0.0393 (8)	0.0377 (8)	−0.0060 (6)	0.0040 (6)	0.0011 (6)
C7	0.0486 (9)	0.0449 (10)	0.0551 (10)	0.0079 (7)	0.0114 (8)	0.0048 (8)
C8	0.0509 (10)	0.0567 (10)	0.0418 (9)	−0.0005 (8)	0.0154 (7)	0.0067 (8)

### Geometric parameters (Å, °)

**Table:**

S1—C3	1.7683 (16)	C2—C3	1.395 (2)
S1—S2	2.0986 (6)	C2—H2	0.9300
S2—O2	1.4358 (13)	C3—C4	1.413 (2)
S2—O1	1.4428 (13)	C4—C5	1.410 (2)
S2—O3	1.4627 (13)	C5—C6	1.373 (2)
N1—C4	1.360 (2)	C5—H5	0.9300
N1—H11N	0.87 (3)	C6—H6	0.9300
N1—H21N	0.83 (3)	C7—H7A	0.9600
N2—C1	1.4805 (19)	C7—H7B	0.9600
N2—C7	1.491 (2)	C7—H7C	0.9600
N2—C8	1.501 (2)	C8—H8A	0.9600
N2—H12N	0.88 (2)	C8—H8B	0.9600
C1—C2	1.375 (2)	C8—H8C	0.9600
C1—C6	1.391 (2)		
			
C3—S1—S2	100.49 (5)	C2—C3—S1	118.90 (13)
O2—S2—O1	116.10 (8)	C4—C3—S1	120.36 (12)
O2—S2—O3	111.22 (8)	N1—C4—C5	120.80 (16)
O1—S2—O3	112.69 (8)	N1—C4—C3	121.86 (16)
O2—S2—S1	109.13 (7)	C5—C4—C3	117.32 (15)
O1—S2—S1	100.93 (6)	C6—C5—C4	121.37 (15)
O3—S2—S1	105.69 (6)	C6—C5—H5	119.3
C4—N1—H11N	116.4 (17)	C4—C5—H5	119.3
C4—N1—H21N	120.4 (17)	C5—C6—C1	120.29 (15)
H11N—N1—H21N	121 (2)	C5—C6—H6	119.9
C1—N2—C7	115.00 (13)	C1—C6—H6	119.9
C1—N2—C8	111.61 (13)	N2—C7—H7A	109.5
C7—N2—C8	109.99 (14)	N2—C7—H7B	109.5
C1—N2—H12N	102.0 (13)	H7A—C7—H7B	109.5
C7—N2—H12N	111.7 (13)	N2—C7—H7C	109.5
C8—N2—H12N	105.9 (13)	H7A—C7—H7C	109.5
C2—C1—C6	120.13 (15)	H7B—C7—H7C	109.5
C2—C1—N2	121.91 (14)	N2—C8—H8A	109.5
C6—C1—N2	117.94 (14)	N2—C8—H8B	109.5
C1—C2—C3	120.15 (15)	H8A—C8—H8B	109.5
C1—C2—H2	119.9	N2—C8—H8C	109.5
C3—C2—H2	119.9	H8A—C8—H8C	109.5
C2—C3—C4	120.73 (15)	H8B—C8—H8C	109.5
			
C3—S1—S2—O2	−61.58 (9)	S2—S1—C3—C2	−88.47 (13)
C3—S1—S2—O1	175.65 (8)	S2—S1—C3—C4	92.94 (13)
C3—S1—S2—O3	58.12 (8)	C2—C3—C4—N1	−177.24 (16)
C7—N2—C1—C2	21.8 (2)	S1—C3—C4—N1	1.3 (2)
C8—N2—C1—C2	−104.36 (18)	C2—C3—C4—C5	1.0 (2)
C7—N2—C1—C6	−159.86 (15)	S1—C3—C4—C5	179.60 (12)
C8—N2—C1—C6	73.96 (18)	N1—C4—C5—C6	178.39 (17)
C6—C1—C2—C3	0.9 (2)	C3—C4—C5—C6	0.1 (2)
N2—C1—C2—C3	179.22 (14)	C4—C5—C6—C1	−0.7 (2)
C1—C2—C3—C4	−1.6 (2)	C2—C1—C6—C5	0.2 (2)
C1—C2—C3—S1	179.85 (12)	N2—C1—C6—C5	−178.16 (14)

### Hydrogen-bond geometry (Å, °)

**Table:**

*D*—H···*A*	*D*—H	H···*A*	*D*···*A*	*D*—H···*A*
N1—H11N···O1^i^	0.87 (3)	2.36 (3)	3.136 (3)	148 (2)
N1—H21N···O1^ii^	0.82 (2)	2.28 (2)	3.010 (2)	148 (2)
N2—H12N···O3^iii^	0.88 (2)	1.89 (2)	2.769 (2)	175 (2)
C5—H5···O3^i^	0.93	2.55	3.376 (2)	148
C8—H8A···O2^iv^	0.96	2.41	3.209 (3)	141

Symmetry codes: (i) *x*, *y*−1, *z*; (ii) −*x*+2, −*y*, −*z*+1; (iii) −*x*+1, −*y*, −*z*+1; (iv) *x*−1/2, −*y*−1/2, *z*+1/2.
